# Moving on: Transition experiences of young adults with chronic pain

**DOI:** 10.1080/24740527.2019.1587707

**Published:** 2019-04-26

**Authors:** Andrea Higginson, Paula Forgeron, Denise Harrison, G. Allen Finley, Bruce D. Dick

**Affiliations:** a School of Nursing, Faculty of Health Sciences, University of Ottawa, Ottawa, Ontario, Canada; b Department of Anaesthesia, Dalhousie University, Halifax, Nova Scotia, Canada; c Children's Hospital of Eastern Ontario, Ottawa, Canada; d Department of Anesthesia & Psychology, Dalhousie University, Halifax, Nova Scotia, Canada; e Centre for Pediatric Pain Research, IWK Health Centre, Canada; f Depts. of Anesthesiology and Pain Medicine, Psychiatry & Pediatrics, Faculties of Medicine and Dentistry & Rehabilitation Medicine, University of Alberta, Edmonton, Alberta, Canada

**Keywords:** transition, chronic pain, young adults, adolescents, young people

## Abstract

**Purpose**: The purpose of this study was to explore the transition experience of young adults with chronic pain in Canada from the pediatric health care setting to the adult health care setting.

**Materials and Methods**: A qualitative descriptive approach using semistructured interviews was used to capture the transition experiences of young people with chronic pain who have recently transferred from the pediatric setting to the adult health care setting. Participants were recruited from west, central, and the east coast of Canada to situate the findings within the context of Canada. Interviews were transcribed and analyzed using qualitative inductive content analysis.

**Results**: Nine participants were interviewed, three from each part of Canada (west, central, and east). Five common categories were determined to describe the transition experience of young adults with chronic pain which include (1) independence (I can do it, maybe?), (2) pain trajectory (stress and pain along for the ride), (3) social support networks (need a shoulder to lean on), (4) parental support (obviously they are there), and (5) collaborative systems (the bridge).

**Conclusion**: Young people with chronic pain experience unique challenges when faced with transitioning to the adult health care setting. Supporting the young person and his or her family in preparation and readiness and collaboration between the pediatric and adult health care settings are essential to ensure a smooth transition and avoid negative transition outcomes. Further research is needed to determine the best ways to prepare young people for transition and the care activities required in both pediatric and adult health care settings to improve pain-related outcomes posttransition.

## RÉSUMÉ

**But**: Étudier l’expérience de transition des jeunes adultes souffrant de douleur chronique au Canada d’un établissement de soins pédiatriques à un établissement de soins pour adultes.

**Matériel et méthodes**: Une approche descriptive qualitative ayant recours à des entrevues semi-structurées pour recueillir les expériences de transition de jeunes souffrant de douleur chronique qui ont récemment été transférés d’un établissement de soins pédiatriquse à un établissement de soins pour adultes. Les participants ont été recrutés sur la côte Est, dans l’Ouest et au centre du Canada pour que les résultats puissent être situés dans le contexte du Canada. Les entrevues ont été transcrites et analysées à l’aide d’une analyse de contenu inductive qualitative.

**Résultats**: Neuf participants ont été interviewés, soit trois de chaque partie du Canada (ouest, centre et est). Cinq catégories communes ont été définies pour décrire l’expérience de transition des jeunes adultes souffrant de douleur chronique, soit *i) l’indépendance; je suis capable, peut-être?* ii) *la trajectoire de la douleur: stress et douleur tout au long du parcours* iii) *les réseaux de soutien social; le besoin d’une épaule sur laquelle s’appuyer iv) le soutien parental; de toute évidence, ils sont présents* et *v) les systèmes de collaboration; le pont.*

**Conclusion**: Les jeunes souffrant de douleur chronique font face à des défis particuliers lorsqu’ils doivent faire la transition vers des établissements de soins de santé pour adultes. Il est essential de soutenir les jeunes et leur famille ainsi que d’établir une collaboration entre l’établissement pédiatrique et l’établissement pour adultes, afin que la transition se fasse en douceur et éviter que la transition entraine des effets négatifs. D’autres études sont nécessaires pour déterminer les meilleures façons de préparer les jeunes à la transition et les soins nécessaires dans les deux milieux, pédiatrique et pour adultes, afin d’obtenir de meilleurs résultats en ce qui concerne la douleur post-transition.

## Introduction

Chronic pain is reported to affect one in five Canadian children, with an estimated 5% to 8% of the adolescent population suffering from pain severe enough to interfere with their quality of life.^1–4^ Furthermore, longitudinal studies suggest that childhood chronic pain predisposes continuation of pain into adulthood as well as the development of new-onset chronic pain.^5^ A significant proportion of adolescents with chronic pain will continue to require health services for pain management into adulthood.^6,7^ Undertreated chronic pain has a negative impact on all aspects of health-related quality of life, including physical (poor sleep, activity limitation), emotional (anxiety, depression), social (peer and family relationships), cognitive function and learning, and role function (absence from school and work).^8^ Considering the impact that chronic pain has on these aspects of life, they may pose unique challenges for young people with chronic pain and their ability to transition into adulthood and into the adult health care system to access treatment. However, little is known about the best practices for this specific population or about the transition experience of this population moving into the adult health care system.

In recent years, transition from pediatric health care to the adult health care system has gained attention, because transition has proven to be a challenge for young people with diverse pediatric chronic illnesses, including congenital heart disease, type I diabetes, and cystic fibrosis.^9–12^ Health care transition is a process that encompasses the affective, behavioral, and cognitive abilities of an adolescent, supported by their family and medical team, to prepare, begin, continue, and complete the transfer from pediatric health care to adult health care.^13,14^ However, for many children with chronic illness, transition to adult health care settings has not always been successful.^15–18^ The Canadian Association of Pediatric Healthcare Centres (now Children’s Healthcare Canada) recently published guidelines to support successful transition for youth with special health care needs.^19^ They define transition as the purposeful, planned movement of adolescents with chronic medical conditions from child-centered to adult-oriented health that is supported by pediatric settings, a coordinated transfer of care, and secure attachment to adult care. The aim of the guidelines was to influence transition at the person and clinical care levels, provide a framework for a supportive process for the transition, and identify collaborative processes, tools, and resources for all stakeholders in transition.^19^ Considering that the guidelines have only recently been developed, it is difficult to determine whether they have had an impact on the transition of adolescents with chronic pain.

For some young people with chronic pain, transfer to adult health care services is unavoidable. In other populations with chronic conditions, ensuring that young people are ready to transfer (health care transition readiness) is linked to successful transition.^19,20^ It is not only the health care transition readiness of a young person that requires assessment but their emotional, behavioral, and cognitive capacities to manage in a new health care environment. Thus, comprehensive ongoing assessment of young people’s supports and their transition readiness is needed to ensure that their transfer is seamless, appropriate, and successful.^20^ Elements that impact health care transition readiness include age, disease or condition, participation in transition clinics, anxiety, and the patient’s and his or her parents’ confidence in the adult health care provider.^21^

Several studies examining patients with chronic illnesses and their transfer experience to adult health care have been conducted.^10,12,18,22,23^ Although these studies provide some insights into concerns and recommended preparation for transition for young people with chronic illnesses, the chronic pain population may have different experiences. This population may have unique challenges that have significant potential to impact health-related outcomes. Improving understanding of the transition experience for young people with chronic pain will provide insights into strategies to ensure a smooth transfer and decrease potential negative outcomes in this population. Thus, the purpose of this study is to understand the transition experience of young people with chronic pain who have recently moved into the adult health care system.

## Material and methods

Due to the paucity of literature on the transition experience of young people with chronic pain, a qualitative approach using semistructured interviews was employed. A qualitative descriptive design, as described by Sandelowski,^25^ was implemented to capture the experience of the participants in detail. This methodology focuses on the experiences of patients and/or their perceptions of patient–professional interactions and the organization of the health care system.^24^ Qualitative descriptive studies offer a comprehensive summary of an event in everyday terms^25^ and can provide insights into understudied phenomena.^26^ The exploration of the experience of young people with chronic pain who have transferred from the pediatric to the adult health care setting will illuminate their individual realities, which will provide helpful insights into the needs of young people to support successful transition.

### Sample and setting

Young people who transitioned from pediatric to adult chronic pain settings between 2014 and 2017 were invited to participate. Recruitment was done through community outreach in an urban Ontario city (e.g., community posters) and online recruitment approaches (email listserves, the University of Ottawa Pain Hub website posting) across Canada. Targeted recruitment was also conducted through two pediatric chronic pain clinics, one in western Canada and one in eastern Canada. Clinicians in these two clinics sent invitation letters to eligible participants who had transferred from their clinics to adult health care services for ongoing management of their chronic pain.

The inclusion criteria for the study were that participants must have received care in the pediatric health care system for their chronic pain; continue to need treatment for their chronic pain; be aged between 16 and 23 years (pediatric centers in Canada transfer patients at different ages, typically 16 years of age and older in Nova Scotia to l8 years of age and older in Alberta and Ontario); have transitioned within the past 3 years; and be able to speak and read English or French. Exclusion criteria included those with developmental delay and those experiencing pain from a life-limiting illness.

Purposeful sampling techniques were used in the recruitment process and are advocated for qualitative descriptive studies^25,27^ because the participants need to have experience with the phenomena under study. The sample size in qualitative studies is not predetermined but is based on the depth and richness of the data, which is influenced by the scope of the study, nature of the topic, quality of the data obtained, and study design.^28^ Sample sizes in qualitative studies collecting data through individual interviews can range from one to 15 participants, with six participants being suggested as adequate.^29^ Interviews began as soon as the first person was recruited, with data analysis occurring throughout, providing an iterative process to determine the sample size of nine participants.

### Data collection

Participants were interviewed for the study using semistructured interviews conducted in person or over Skype. An interview guide was used that included a list of topics to be covered during the interview^27^ and moved from closed-ended questions to open-ended individual questions.^25^ The closed-ended questions were used to capture demographic information (i.e., age of the onset of pain, age they were transitionned, current age, where they receive their pain care). The open-ended questions provided participants the opportunity to emphasize the important issues from their transition experience. The interview guide was informed by Meleis’ transition theory, as expectations and one's abilities create a period of vulnerability.^30^ The adolescent with chronic pain experiences two different types of transition simultaneously: developmental and situational. The developmental transition occurs as he or she moves from adolescence to adulthood and, according to Meleis, this is marked by increased self-identity and growing independence.^31^ The situational transition is a result of the transition that takes place in individual roles and relationships; for example, the change in roles of the child in the home to an independent adult and the impact that this has on the family relationships.^30^ In addition, situational transition includes geographical changes, such as changes in where a young person lives when he or she moves away from home or the change in team membership among those from whom the young person receives health care.^30^ The open-ended nature of the questions allowed the interview format to be flexible and facilitate an open discussion about the transition experiences of importance to the participant. The interviewer also wrote field notes after each interview to capture impressions and observations of the interviews. The interviews were audio recorded and transcribed for analysis.

## Data analysis

Qualitative inductive content analysis was used to analyze the data. This method allowed for a condensed yet broad description of a particular phenomenon, through the use of concepts and categories.^32^ The knowledge generated from qualitative content analysis is based on the participants’ unique perspectives and grounded in the actual data.^32^ The benefit of using this approach was that the insights on transition by participants were obtained without imposing preconceived categories or perspectives. Although Meleis’s theory of transition (along with the literature on health care transition) informed the development of the interview guide, it was not used as a framework in the inductive content analysis because categories were not determined a priori.^31^ An inductive approach moves from the specific meaning to the general categories to aid in understanding of the phenomena using three steps: open coding, creating categories, and abstraction.^32^

During open coding, the researcher reads through the transcripts and identifies headings (codes) and makes notes directly on the document. In this study, the principal investigator (A.H.) highlighted and identified exact words in the text that captured the key concepts and thoughts of the participants^33^ using as many codes as necessary.^32^ After the first two transcripts had been coded, a code list was developed and reviewed with the second author (P.F.). This list was used to help code the remaining interviews. If new codes had emerged in subsequent interviews the codes would have been added; however, no new codes emerged after the coding of the first two transcripts. Once the codes were applied, the data were categorized, which is a key activity of qualitative content analysis.^34^ In this study, codes were collapsed into categories by determining how a broader descriptive level of content fit the data.^34^ The categories (and the direct quotes that act as exemplars to support these categories) were further refined to reflect patterns of behavior that were relevant to the study as described in the interviews.^27^

The researcher then used abstraction to compile the data. Abstraction is defined as general descriptions of the research topic through generating main categories.^32^ Through an analysis of how the categories string together, an understanding of the experience of transition for young people with chronic pain was described. Each main category name was constructed from the content characteristics of data.^32^ All authors then reviewed the identified main categories to ensure that they were grounded in the data.

### Rigor

The most commonly used criteria to assess rigor in qualitative research are credibility, dependability, conformability, and transferability, as proposed by Lincoln and Guba.^35^ Credibility is described as the degree of confidence in the findings that the researcher inspires in the reader. Strategies to improve credibility include prolonged engagement, persistent observation, and triangulation.^36^ Dependability is achieved when the researcher accounts for the alterations in the researchers’ decisions during the analysis process.^36^ This was achieved through peer debriefing with the second author. Transferability refers to the extent to which findings may have application to different settings^34,37^ and is enhanced by “thick description.”^38,39^ In this study, thick description included a description of the chronic pain clinical setting (both the pediatric chronic pain setting that participants once attended and the adult chronic pain setting that they now attend), the unique participant characteristics (e.g., age of pain onset, type of community where they live [rural or urban], gender, present age), context for the interview (e.g., Skype or in person, providing observation of facial expression as well as tone of voice), data collection approach (e.g., semistructured interviews), and data analysis (e.g., inductive content analysis).^34^ Additionally, a rich description of the participants’ experiences of transition with the use of exemplar quotes to support the main categories offer further detail to enable the transferability of the findings. Because this was a multisite study, the transferability was further enhanced because the participants’ experiences were collected from participants located in diverse regions across Canada.

### Ethical considerations

Ethics approval was obtained through the research ethics boards at the following institutions: IWK Health Centre, Halifax, Nova Scotia; University of Alberta, Edmonton, Alberta; and the University of Ottawa, Ottawa, Ontario. Participation in the study was voluntary and verbal consent was obtained and transcribed on the date of the interview. Aliases were given to each participant as part of the confidentiality technique.

## Results

### Demographics

Nine young people participated in the study, including eight women, and one man, ranging in ages from 18 to 23. Two participated in person and seven by Skype. Eight were receiving care for their chronic pain in the adult health care setting and one was no longer receiving care despite still experiencing chronic pain (see Table 1). The young people experienced a variety of chronic pain conditions: one had concussions, two had complex regional pain syndrome, one had musculoskeletal pain the lower limbs, one had sickle cell anemia, one had endometriosis and soft tissue damage, one had juvenile rheumatoid arthritis, one had avascular necrosis, and another suffered from daily headaches. They reported a wide range of transition experiences, including positive experiences (successfully receiving care for their chronic pain in the adult health care setting) to negative experiences (long delays to be seen in the adult health care setting for pain management). Although the participants discussed their experiences of transitioning between the health care environments, each story encompassed developmental transition experiences, which were interwoven with their health care transition.

**Table 1.** Demographics of participants.

Alias	Current age	Age of diagnosis	University/working	Method of interview
Stephanie	23	16	Completed/working	Face-to-face
Lisa	21	12	University	Skype
Brenda	20	16	University	Face-to-face
Jen	18	17	University	Skype
Melissa	23	15	Completed/working	Skype
Betty	21	15	Not in school/not working	Skype
Julie	20	16	Not in school/not working	Skype
Sally	21	16	University	Skype
Charles	19	12	University	Phone

Six of the nine participants had moved to a different city in order to further their postsecondary education. This situation created another level of complexity for their transition because they had to access chronic pain care in a city that differed from where their pediatric providers were located and not all pediatric pain clinicians knew a chronic pain adult health care provider to transfer them to in their new locations. For some of the young people, this meant that they were transferred to either their previous or a new general practitioner with or without referral to a specific adult chronic pain specialist. For the three participants who remained in the same city where they accessed their pediatric pain care, their transition experience was voiced as being more successful and straightforward, because their pediatric clinicians knew the adult clinicians to whom they were being transferred.

From the analysis of participants’ collective experiences, five distinct major categories emerged. Although these categories are distinct, there is also overlap between the categories because it is not possible to delineate one’s life experiences into isolated events. Nevertheless, what follows is a discussion of the five major categories, which include (1) independence (I can do it maybe?); (2) pain trajectory (stress and pain along for the ride); (3) social support networks (need a shoulder to lean on); (4) parental support (obviously they are there); and (5) collaborative systems (the bridge).

### Independence: I can do it maybe?

Many participants expressed that when they were getting ready to transition into the adult health care system they were not concerned about that change. They felt confident and ready to go to university and to move to the next chapter of their lives. From a developmental perspective, feeling confident and excited about the next stage of life (e.g., attending postsecondary education, moving away from home, starting a career) should be viewed positively. However, in terms of their health care transition, this confidence was perhaps overly optimistic because they were not fully cognizant of potential limitations or needs due to their chronic pain. For some participants, the management of their chronic pain was not a concern as they began university trying to live a life like their peers. Similar to their peers without chronic pain, they started the semester ready to participate in all sorts of activities; however, as time went on, they had the added challenge of finding services and managing their chronic pain in new environments and without some of their previous supports. Betty expressed excitement about moving and began the year with high hopes. However, she predicted being able to achieve more than what she was actually able to and, as the semester progressed, she realized that her chronic pain created limitations.

[University] was a good experience and it started giving me life again ’cause I think I was in a pretty depressive state, so as soon as I got there, I signed up for everything and over did it and burnt out pretty quickly.

The majority of the participants voiced that when they were leaving the pediatric pain clinic, they recall being instilled with the notion that accessing health care for their chronic pain management would be straightforward even when moving away to university. Although they had discussions about transition with their pediatric chronic pain teams, they reported that they were not really clear about what transition truly meant. When Jen was asked what her transition assessment process consisted of before leaving the pediatric chronic pain clinic, she could not specifically identify what was done to prepare her for transition. “They didn’t ever have me take a survey or anything like that, they just kind of said, do you feel ready? And I said well, I’m as ready as I can feel.” This notion of access to health care being straightforward once they transferred to adult care created a sense of confidence that everything would work itself out. Participants shared that they did not realize the amount of effort that would be required to locate and access chronic pain management services within the adult health care system. All still believed that all of their care needs would be coordinated for them as it was in their pediatric chronic pain clinics. The foundation of their confidence in the coordination of their future care is uncertain. However, given the positive experiences in coordinated care in their pediatric chronic pain clinics, these young people may have simply presumed that this would continue. Nevertheless, they experienced challenges in health care access that left them feeling less than confident in their abilities to be independent in managing their chronic pain and accessing care to support their care needs. Brenda shared that when she went away to university she believed that the transition to the adult chronic pain team in her new city would have been established and that she would seamlessly access care. However, at the time of the interview, she had been at university for 3 years and was still waiting to have an appointment with the chronic pain team. Furthermore, Brenda believed that her pediatric chronic pain team contributed to her confidence by not preparing her for how challenging it could be once she arrived at university.

Honestly no [preparation for transition]. Like not now, I may have felt [then I was ready for transition] I was leaving because it had been two years [attending pediatric chronic pain services] and it’s just like, “Oh, this is great it [city she was moving to] has many services there,” but it appears that there is no services and no support, I’m still in this, “Like, but, wait … [where do I get help]?”

### Pain trajectory: Stress and pain along for the ride

Along with the transition in health care, the participants were transitioning to their next stage of life. Like other people of similar ages, they were moving on after high school to postsecondary education or employment, with most moving away from their home cities. Like their healthy counterparts, these young people experienced stress and anxiety about leaving home and beginning their university education. However, unlike their healthy counterparts, increased stress often exacerbated of their chronic pain. Furthermore, unlike their peers, their stress was not isolated to issues surrounding the typical university experience but also included concerns about seeking care if their pain became unmanageable in their new environments. Lisa expressed experiencing concerns about being in a new city and not being able to access the same pediatric providers after she moved. However, Lisa’s experience was unique because she was treated for chronic pain in her hometown through videoconferencing. Fortunately for Lisa, this experience of changing cities did not impact her access to regular appointments with the pediatric or adult chronic pain team. Furthermore, Lisa continued to have access to her pediatric team through her first year of university, which she expressed gratitude for, because she believed that this led to a successful transition.

Oh, yeah, absolutely, and I mean she was fabulous in that way [the support of the pediatric chronic pain nurse], she was always just an e-mail away but I think where my anxiety came from was that I no longer had, like, the physio clinic that I had gone for years behind me and I didn’t know necessarily how to get those resources at first and on top you’re moving away for school, you know, university, and it’s all a new experience and all of those wonderful things. So, hum, there was actually my pediatric team, it’s like very like, confusing at the time because they overlap so much, so my pediatric chronic pain team, at the time ended up continuing to work with me, because I was nervous for school hum, they help me connect with my advisor [student access service advisor].

Lisa experienced a well-supported transition; she identified that she was able to contact the pediatric and adult chronic pain teams as she needed. Furthermore, she identified that she was able to manage her own chronic pain and that this was something that the pediatric team had worked with her to accomplish. She still experienced increased stress during her transition, but her supportive transition helped her manage, because she had ongoing access to familiar expert pain care. Similarly, Stephanie noted that although her stress about starting university caused an increase in her chronic pain, she was able to manage. She transitioned to the adult pain clinic in her hometown, and although she had to commute home for appointments, she had access to expert pain care. She also was starting to develop strategies to manage between appointments. “Stress of going away definitely made my headaches worse, but I sort of learned to deal with them on my own.”

Unfortunately, those who voiced not having an identified chronic pain clinician in the adult health care system experienced worse outcomes. Julie did not have health care services in place when she left the pediatric chronic pain team and she had not been transitioned to an adult chronic pain clinic. This less than ideal situation resulted in stress, which she felt contributed to making her pain worse. “It has gotten worse, like, I said now I see more inflammation where my knee gets red hot to touch like through my clothing.” It may be that Julie was not prepared to manage her pain independently before leaving the pediatric chronic pain clinic. She was trying to finish her high school credits and was having difficulty going to work, which further exacerbated her stress and pain levels, creating a vicious circle with no adult chronic pain clinicians available to help her.

Although the majority of the participants spoke of experiencing increased stress when starting university and discussed how this stress exacerbated their pain, for a few participants, the opposite was experienced—increases in their pain intensity exacerbated their stress and anxiety. Melissa explained how during class when her pain would increase, her anxiety would also increase, and this experience almost became unmanageable.

I just work through it, I sat there in a whole lot of pain trying to do the work and trying to get stuff done and I was miserable like, the anxiety shot right up. I almost had to go onto pills for that [anxiety].

The interplay between pain and stress or anxiety is known, but the experiences of these participants illustrates that transition encompasses stressful events associated with transferring health care settings as well as their developmental transition.

### Social support networks: A shoulder to lean on

The need for peer support and being able to form peer networks are part of adolescent and young persons’ lives. This was true for the participants in this study as well; however, the majority of the participants voiced that creating friendships and lasting relationships was difficult as a result of their chronic pain. Jen acknowledged the importance of having a network of friends but identified this as a challenge for people with chronic pain. “I think that’s really important, to have a social network, I know it’s really difficult for people with chronic pain to get out and have a social network.” When these participants were challenged to garner support from peers and build a reliable peer network, it had negative consequences on their health care and developmental transition outcomes. Fear of judgment and a perceived lack of understanding of chronic pain were cited as reasons why many of these participants did not share their chronic pain condition with peers. Brenda identified that she does not share that she has chronic pain with her new friends at university because she has faced negative reactions from others in the past, which left her fearful of disclosure.

I do not personally tell people until I feel I have a trusting relationship with them, ’cause, again, I don’t want to tell them and then have them hurt me ’cause it can flip and then they could just react negatively, so I want to be able to build a relationship and trust them before I came out and say it.

Most participants were either starting postsecondary education or careers and therefore did not feel comfortable disclosing their condition to others, which created barriers to obtaining the social support they needed from these new friends. This is concerning because many of the participants moved away from home at the same time they were transitioning to adult health care services for their pain management needs, so when they may have needed more support from friends they were potentially receiving less. Furthermore, due to functional limitations associated with their chronic pain, if new friends do not understand the reason for limitations, the lack of disclosure may become a barrier to establishing new relationships. Charles stated that he was able to make new friends; however, his new friends did not know that he has chronic pain, so it was difficult to understand the sorts of pain-related social support he was able to garner from them. “Yeah, I made a, like four more friends from different classes that I rely on. Oh no, they don’t [know that I have chronic pain].”

Even when these young people were able to establish new friendships, some still needed other forms of social support. Melissa found that although she had made new friends, she had a difficult time relating to them about her chronic pain, because they did not share a chronic pain or chronic illness experience. It appears that negative social experiences in high school made participants perceive their new friends as unable to provide pain-related social support. Therefore, some participants, like Melissa, tried to find peer support from others with chronic pain in the form of a group.

This past appointment, … I asked again [about a pain support group] because I have friends now, I have four really good friends, which is more than enough for me, but none of them have the same problems that I do, they don’t even have allergies.

Several of the participants reported that they sought formal peer support groups to cope with their chronic pain but that they encountered challenges in finding the right group for them. Most of the participants interested in a peer support group wanted to attend a group of their similar-aged peers—not adults who were considerably older—because hearing about the continued struggles of older adults with chronic pain was discouraging and key personal issues are often considerably different between these age groups. Yet, these age-specific chronic pain support groups did not seem to exist.

### Parental support: Obviously they are there

Not surprising, the participants in this study consistently talked about the role of their parents in their transition process. All of the participants talked of how their parents played a role in seeking treatment for their chronic pain condition while in the pediatric health care system. However, for many of the participants, this role continued when they transferred into the adult health care system, meaning that many of these young people continued to be dependent on their parents’ active involvement in order to access care. Stephanie recollected that had she not relied on her mother to advocate for her within the adult health care system she would not have accessed the resources she needed. “I feel like if I didn’t have my mom as a connection then I would be lost.” The need to rely on their parents to advocate on their behalf was also highlighted by Jen and echoed by others:

They [parents] just really helped with not necessarily pushing but kept on asking the doctors at the pain center to make sure that the referral went through so I could see the specialists here as soon as I got here.

This perhaps reflects that most of these young people may not feel completely confident in their abilities to advocate for themselves at the time that they transitioned. Continued reliance on their parents (mostly their mothers) may contribute to young people not developing the skills and knowledge needed to negotiate the adult health care system. Many participants had the expectation that their parents would always be there to provide support to ensure that their chronic pain was well managed. It is unclear whether relying on their parents is any different for young people with chronic pain compared to their healthy peers but the reasons and outcomes may be different. Melissa expressed that while living at home, she relied on her mother to provide her with the health care support she needed, because she did not feel capable of doing it herself. Melissa lived at home with her mother while attending university and, at the time of the interview, continued to live there since she had graduated and was working part-time. “Sometimes she had to pull me off the couch to get me to the bedroom or offer to get me my pills when they’re three feet away.” For some of the participants (but not all), their continued reliance on their mothers to take the lead in their chronic pain needs may impede their transition into adulthood. Charles stated that his mother managed his chronic pain needs and that he viewed this as her role, not a shared responsibility. “She’d deal with them [medication]. I have some right now, she puts these pills in containers and she gets them for me every day.” Clearly there was a range in how much participants relied on their parents for help with their chronic pain management, from minimal reliance to total reliance.

### Collaborative systems: The bridge

The health care transition journeys of these participants highlighted the critical role of collaboration among the pediatric and adult health care providers in bringing about a successful health care transition for adolescents and young people with chronic pain. The range of experiences demonstrates the relatively smooth path that some of the participants experienced when collaboration was high and the rocky path when collaboration was low. For Lisa, transferring from the pediatric to the adult health care team was characterized by teamwork in coordinating her transition activities.

We went over together [with the pediatric nurse] so she actually helped facilitate it, so it was gradual. It was in terms, this is your last day in the pediatric clinic. It wasn’t that I was at any point lacking care because the adult clinic had come in, they started working with my pediatric team to kind of bridge the gap.

It is possible that Lisa’s successful transition could be attributed to the physical proximity of the two teams, because they were both in the same city where she was already receiving care. For other participants, such a coordinated team approach was not the case. Betty explained that a meeting between the pediatric and the adult health care team had been discussed but never took place.

No, they talked about having that, like having both come together [pediatric and adult health care teams] but from what I recall it didn’t happen. I just kind of went straight into the adult system.

Some of the participants were referred back to their general practitioners in their hometowns, which caused additional challenges in accessing pain care when they moved to a new city for university or careers. Sally expressed her distress, saying, “I was kind of worried because I didn’t have a doctor up here” and admitting that she really did not know who to turn to help her manage her chronic pain in the city where she was attending university.

Transferring participants back to their general practitioners rather than to an adult pain clinic was not uncommon. The integration of one’s primary practitioner along with specialist care is arguably an appropriate approach to provide more comprehensive care. However, these participants were surprised by the way in which their pain management care was referred back to their primary care provider, because this was perceived as different from when they accessed care in the pediatric health care system.

Complicating the situation for young people who are transferred to their primary care provider is that the range of knowledge and skills needed to effectively manage chronic pain varies greatly among primary health care providers. This range of pain expertise can pose complications for the newly transitioned young people. Betty experienced unexpected complications, potentially as a result of her well-meaning primary care provider not having pain expertise.

At the clinic at the university, a doctor there had agreed to be my temporary physician while I was at school and so he had contacted my chronic pain doctor and said that this was alright to put me on fentanyl, I don’t know why I didn’t think of it cause I was already on pain killers, and because it’s long lasting he thought it would be helpful, but they just put me on too high of a dose too early and that’s why I left university, I couldn’t stay awake, so I would sleep through, I wouldn’t wake up from alarms, my RA would have to, like, people would be banging on my door to wake me up.

Betty’s experience speaks to the need to not only ensure that young people are transferred to health care providers who have foundational chronic pain management knowledge but that they themselves are knowledgeable about their treatments. Despite collaborating with a chronic pain physician, it appears as though Betty was not instructed to stop her other opioid analgesia, which may have contributed to the degree of her side effects. It is possible that adult health care providers assume that these young people have an understanding of their pain management treatments. The significance of poor management cannot be understated because not only was Betty at risk for safety issues due to oversedation but she had missed classes and withdrew from her university education.

Some participants were transferred solely to a primary health care provider. This situation occurred either because there were no adult chronic pain providers where they lived or because the pediatric chronic pain team did not have an established relationship with the adult chronic pain team in the city where the participant was moving. For example, Brenda was never transferred to an adult chronic pain health care team once she left for university. Although she was placed on the waiting list as a new patient to be seen in the adult chronic pain clinic in her new city, 3 years later, at the time of interview, she was still waiting to be seen.

I see a general physician that I’ve seen since I was a kid I mean in my hometown. I’ve been trying to get into the adult chronic pain center here. I have two letters sent to me saying that the nurse will be phoning to set up time for a referral.

Furthermore, Brenda had to take the initiative to send in the referral letter from the pediatric team to the chronic pain team in the adult system in the city where she went to university. Though she had had the same primary care physician since childhood she noted that this doctor did not fully understand her history or her pain management strategies. This suggests that her primary care physician was not an integral member of her pediatric chronic pain team, which now meant that she was accessing her chronic pain care from a physician who did not have adequate knowledge of chronic pain or a full understanding of her medical history.

## Discussion

Through the sharing of transition experiences by the participants in this study it became clear that these young people with chronic pain share some challenges with other populations of young people with chronic conditions but also experience some unique challenges. As adolescents preparing for transition, the participants expressed being excited about moving on to the next chapter in their lives, such as leaving home for postsecondary education. Many of the participants felt a sense of confidence in initially leaving the pediatric setting. This confidence coupled with their inability to attain needed health care services suggests that at the time of transfer, they may not have developed the necessary knowledge and skills to manage the adult health care system and thus may not have been ready for transition.

At the time of transfer, the participants of this study were all in the developmental phase of emerging adulthood. According to Arnett, emerging adulthood is defined and described as a process during which young people are learning to develop their individuality and is represented by becoming independent from others (especially from parents) and learning to be a self-sufficient individual, by becoming responsible for one’s self and making independent decisions.^40^ Thus, health care transition comes at a time when most young people are still developing the abilities to be self-sufficient individuals, despite many of them needing these abilities to access care. The participants in this study are no exception because they identified the necessity of relying on their parents to help them secure the care they needed. Adult health care systems claim to be patient-centered, and adult patients need to manage their conditions and navigate the health care system themselves; however, it may be unreasonable to expect emerging adults to do this independently without at least initial support from their parents.

Contrary to the literature on transition, the majority of these participants did not report experiencing heightened levels of anxiety leading up to the transfer to adult health care services.^21,22,41^ The reasons for this are not clear but it may be that the participants in this study believed that accessing pain care in the adult health care setting would be similar to their pediatric experience. Due to the limited number of chronic pain clinics in Canada, geographic barriers may pose a challenge for pediatric practitioners to transition them to adult chronic pain clinics.^42^ As seen in this study, lack of chronic pain clinics resulted in some young people with chronic pain being transitioned to a general practitioner who may not have the expertise to provide them with the care that they require. General practitioners have identified experiencing challenges in treating their patients with chronic pain, including understandable reservations about prescribing medications for chronic pain management.^42^ More research is needed to determine the best ways to help general practitioners improve their knowledge and skills in caring for young people with chronic pain and be integrated into the transition process. In Canada there is inadequate access to regional multidisciplinary chronic pain management centers. Collaboration and shared care in transition needs to include the young person’s primary care providers earlier in their care. Collaborating more formally with general practitioners when adolescents with chronic pain are accessing care from a pediatric chronic pain team may result in family general practitioners having a better understanding of chronic pain treatment for young people, which may improve their transition and decrease negative outcomes.

By mid-adolescence, most young people identify their best friend as their main source of social support.^43,44^ However, adolescents with chronic pain experience multiple challenges in peer relationships and friendships^45^ and many report experiencing friendship loss since the onset of pain.^46^ Peer support has been identified as having a positive impact on transition for young adults with chronic illnesses.^47^ However, for these young people with chronic pain, establishing a reliable peer support network proved to be a challenge. Some participants in this study identified that they found it difficult to create lasting friendships during high school, and this challenge carried over as they entered the phase of emerging adulthood when attempting to create social networks at university or work. When unable to establish and maintain friendships, young people with chronic pain who are transitioning to another developmental stage in their lives may not be able to secure needed social support to help them cope with their chronic pain. The role of strong friendships, perhaps as a predictor of transition readiness, warrants further investigation.

Research suggests that peer support from a person with a similar chronic illness can be helpful with the transition process,^47^ and some of the participants in this study sought peer support groups as a strategy to cope with the lack of social support they perceived from their friends. However, not all adult health care services offer age-specific peer support groups and, as highlighted by participants in this study, one has to question whether peer support by people considerably older offers the same benefits as peer support by those closer to one’s own age. Research is needed to understand what kind of peer supports may be helpful in the context of transition for young people with chronic pain.

Parents play a positive supportive role in the transition process^10,17,18,48^ and are key in helping their children develop decision-making skills.^16^ Learning decision-making skills in a supportive setting with the encouragement of parents is linked to positive transition outcomes.^17^ The majority of the participants in this study reported that their parents played a supportive role in their transition to the adult health care setting. However, some participants still relied on their parents to organize their care (e.g., organize their medications) and others spoke of regressing to the point where they would get their parents to help them mobilize. The transition process encompasses not only the transition between clinicians but also the transition between the role of the young adult and the parent in managing health care.^17,49^ This may be more difficult for parents of young people with chronic pain. Parental characteristics such as emotional functioning and associated behaviors, as well as medical history (chronic pain), have been shown to negatively impact not only the child’s perception of and response to pain but also their developmental trajectory.^50^ Specifically, the relationship between adolescents with chronic pain and their parents has been shown to decrease an adolescent’s autonomy from his or her parents both emotionally and in the ability to make independent decisions.^50^ Challenges in the process of transition into adulthood also negatively influence the ability of an adolescent with chronic pain to function autonomously in the adult health care setting. Furthermore, higher levels of parent involvement in chronic pain–related disease management activities are associated with increased levels of adolescent disability.^50,51^ Both of these findings suggest that clinicians in the pediatric and adult chronic pain settings may need to help parents and adolescents to gradually shift care. Little is known about the transition process from the perspective of parents whose child has chronic pain and thus transition strategies to support parents are not specific to the chronic pain context and warrant study.

Open collaboration between pediatric health care clinicians and adult health care clinicians prior to transition is linked to improved outcomes.^41^ Strategies such as a joint meeting between the patient, family, and a member from both teams provide an opportunity for collaboration between all stakeholders. Collaboration between stakeholders can reduce anxiety, foster a more effective working relationship, and create a more positive transition experience.^41,52^ Research suggests that the closer the relationship between pediatric and adult health care clinicians, the more positive and seamless the transition will be for the young person transitioning.^15,16,18^ Of note, only one participant in this study reported having the opportunity to meet adult health care providers before transitioning, and another was able to receive care from both pediatric and adult chronic pain care providers for a period of time. However, the majority of the participants did not have such a collaborative process during the transfer of their care and these participants experienced significant negative outcomes, including dropping out of university, admission to hospital for pain care, being oversedated on opioids, and not receiving care despite continued chronic pain. Promoting continuity of contact with pediatric health care providers has been found to reduce the risk of long-term disengagement with care for young adults with chronic illnesses requiring transition and to reduce missed appointments in the adult system.^53^ Research into the barriers to providing more collaborative care transition processes is needed to inform the development of strategies to improve transition outcomes for young people with chronic pain.

The main clinical recommendation from this study is that the transition of a young person with chronic pain should be considered and supported by both health care settings. A successful transition to the adult health care system has been noted to be one in which the young person is able to communicate his or her wishes and needs freely with confidence and self-esteem.^54^ This can be supported through clinical practice in pediatrics by providing care to the patient independent of their parents. Seeing the young person alone at least for part of the consultation can influence independent health management.^55^ It is important to provide some independence while in the pediatric setting, because many young people, including those in this study, have identified wanting independence but may not fully understand what this entails.^41^ A young person must learn how to integrate new information, acquire new skills, and simultaneously monitor different factors, including physical symptoms, changes in medication regimens, and appointment scheduling.^17^ By beginning the transition process early, the young person will be better prepared to care for his or her own needs and acquire the skills to problem solve once he or she has transferred to the adult health care setting. Through use of psychometrically validated transition assessment tools, practitioners could determine whether or not a patient is comprehensively managing his or her own care before the transition.^20^ As young people are encouraged to be independent, parents should be encouraged to focus on the young person’s strengths and develop realistic and developmentally appropriate expectations.^56^ Maintaining an open collaboration between the two systems could further support the management of a young person with chronic pain once he or she has been transferred to the adult health care setting. This collaboration would support adult providers, because it has been identified that there is a lack of adult provider understanding of pediatric pain.^57^ Preparing a young person with chronic pain for the transition to the adult health care setting should be a collaborative approach between all stakeholders, including patient, parents, pediatric health care providers, and adult health care providers, to ensure that the process is successful and the young person is acquiring the care that he or she needs.

## Strengths and limitations

The strength of the study was that it captured the experiences of participants across Canada. The nine participants were from western, central, and eastern Canada, and although each participant had a different experience, challenges that they reported were similar. However, there was only one male participant and although this ratio of male to female participants may reflect the prevalence of chronic pain among women compared to men, it is possible that the transition supports may be different for males, which warrants further study. Although this study included females and males across Canada, there were several limitations. First, it is possible that participants did not recollect their experiences with clarity, because for some it had been 3 years since they had transitioned. Secondly, it may be that those who decided to participate are those who faced challenges in their transition process and wanted to tell their stories. However, several of the participants had successful transitions from pediatric to adult chronic pain providers, suggesting that the participants’ experiences are not solely negative.

## Conclusion

Young adults with chronic pain face many challenges when seeking care and moving forward into adulthood. Formalized transition processes are not well established in many centers that serve young people with chronic pain, which can lead to negative outcomes. It is the responsibility of stakeholders (i.e., young people, parents, health care providers) to understand their roles and responsibilities in supporting a successful transition. Starting the transition process early, in partnership with the pediatric and adult health care teams, the general practitioner, parents, and the young person, is paramount to successful transition for young people with chronic pain.
